# Dietary Crocin is Protective in Pancreatic Cancer while Reducing Radiation-Induced Hepatic Oxidative Damage

**DOI:** 10.3390/nu12061901

**Published:** 2020-06-26

**Authors:** Hamid A. Bakshi, Mazhar S Al Zoubi, Hakkim L. Faruck, Alaa A A Aljabali, Firas A. Rabi, Amin A. Hafiz, Khalid M Al-Batanyeh, Bahaa Al-Trad, Prawej Ansari, Mohamed M. Nasef, Nitin B. Charbe, Saurabh Satija, Meenu Mehta, Vijay Mishra, Gaurav Gupta, Salem Abobaker, Poonam Negi, Ibrahim M. Azzouz, Ashref Ali K Dardouri, Harish Dureja, Parteek Prasher, Dinesh K. Chellappan, Kamal Dua, Mateus Webba Da Silva, Mohamed El Tanani, Paul A. McCarron, Murtaza M. Tambuwala

**Affiliations:** 1 School of Pharmacy and Pharmaceutical Science, Ulster University, Coleraine BT52 1SA, UK; mm.webba-da-silva@ulster.ac.uk (M.W.D.S.); p.mccarron@ulster.ac.uk (P.A.M.); 2 Department of Basic Medical Sciences, Faculty of Medicine, Yarmouk University, Irbid 566, Jordan; mszoubi@yu.edu.jo; 3 Department of Mathematics and Sciences, College of Arts and Applied Sciences, Dhofar University, Salalah 211, Oman; 4 Department of Pharmaceutics and Pharmaceutical Technology, Faculty of Pharmacy, Yarmouk University, Irbid 566, Jordan; alaaj@yu.edu.jo; 5 Department of Clinical Sciences, Faculty of Medicine, Yarmouk University, Irbid 21163, Jordan; firas.rabi@yu.edu.jo; 6 Department of Clinical Nutrition, Faculty of Applied Medical Sciences, Umm Al-Qura University, Makkah 21421, Saudi Arabia; aahafiz@uqu.edu.sa; 7 Department of Biological Sciences, Faculty of Science, Yarmouk University, Irbid 566, Jordan; albatynehk@yu.edu.jo (K.M.A.-B.); bahaa.tr@yu.edu.jo (B.A.-T.); 8 School of Biomedical Sciences, Ulster University, Coleraine BT52 1SA, UK; ansari-p@ulster.ac.uk; 9 Department of Pharmacy and Biomedical Sciences, School of Applied Sciences, University of Huddersfield, Queensgate, Huddersfield HD13DH, UK; mohamednasef103@gmail.com; 10 Departamento de Química Orgánica, Facultad de Química y de Farmacia, Pontificia Universidad Católica de Chile, Av. Libertador Bernardo O’Higgins, Santiago 340, Región Metropolitana, Chile; nitinunimi@gmail.com; 11 School of Pharmaceutical Sciences, Lovely Professional University, Phagwara, Punjab 144411, India; saurabh.21958@lpu.co.in (S.S.); meenu.22252@lpu.co.in (M.M.); vijaymishra2@gmail.com (V.M.); 12 Discipline of Pharmacy, Graduate School of Health, University of Technology Sydney, Ultimo, NSW 2007, Australia; kamal.dua@uts.edu.au; 13 School of Pharmacy, Suresh Gyan Vihar University, Jagatpura, Mahal Road, Jaipur, Rajasthan 302017, India; gauravpharma25@gmail.com; 14 Department of Gynecology, European Competence Center for Ovarian Cancer, Campus Virchow, Klinikum Charite-Universitatmedizin Berlin, Augustenburger Platz 1, 13353 Berlin, Germany; salem-nuri.abobaker@charite.de; 15 School of Pharmaceutical Sciences, Shoolini University, Bajhol, Sultanpur, Solan, Himachal Pradesh 173229, India; poonam.546@shooliniuniversity.com; 16 Department of Dermatology, Venerology, and Allergology, Charite-Universitatsmedizin Berlin, Corporate Member of Freie Universitat Berlin, Chariteplatz1, 10117 Berlin, Germany; ibrahim.azzouz@charite.de; 17 Department of Forensic Science, School of Applied Sciences, University of Huddersfield, Huddersfield HD13DH, UK; drdory_02@yahoo.com; 18 Department of Pharmaceutical Sciences, Maharshi Dayanand University, Rohtak, Haryana 124001, India; harishdureja@gmail.com; 19 Department of Chemistry, University of Petroleum & Energy Studies, Dehradun 248007, India; parteekchemistry@gmail.com; 20 Department of Life Sciences, School of Pharmacy, International Medical University, Kuala Lumpur 57000, Malaysia; dinesh_kumar@imu.edu.my; 21 Pharmacological and Diagnostic Research Centre, Faculty of Pharmacy, Al-Ahliyya Amman University, Amman 19328, Jordan; m.tanani@ammanu.edu.jo

**Keywords:** pancreatic cancer, crocin, apoptosis, cell cycle, radiation, hepatic injury

## Abstract

Pancreatic cancer is one of the fatal causes of global cancer-related deaths. Although surgery and chemotherapy are standard treatment options, post-treatment outcomes often end in a poor prognosis. In the present study, we investigated anti-pancreatic cancer and amelioration of radiation-induced oxidative damage by crocin. Crocin is a carotenoid isolated from the dietary herb saffron, a prospect for novel leads as an anti-cancer agent. Crocin significantly reduced cell viability of BXPC3 and Capan-2 by triggering caspase signaling via the downregulation of Bcl-2. It modulated the expression of cell cycle signaling proteins P53, P21, P27, CDK2, c-MYC, Cyt-c and P38. Concomitantly, crocin treatment-induced apoptosis by inducing the release of cytochrome c from mitochondria to cytosol. Microarray analysis of the expression signature of genes induced by crocin showed a substantial number of genes involved in cell signaling pathways and checkpoints (723) are significantly affected by crocin. In mice bearing pancreatic tumors, crocin significantly reduced tumor burden without a change in body weight. Additionally, it showed significant protection against radiation-induced hepatic oxidative damage, reduced the levels of hepatic toxicity and preserved liver morphology. These findings indicate that crocin has a potential role in the treatment, prevention and management of pancreatic cancer.

## 1. Introduction

Pancreatic cancer has become the seventh-largest cause of cancer-related death by 2020 [1], with an expected 5-year survival rate of approximately 8% [2]. It is the seventh leading cause of cancer-related mortality, causing over 300,000 deaths per year [3]. Surgical resection is the treatment of choice, but only a small minority of patients have resectable disease at the time of initial diagnosis [4]. Post-surgical recurrence has remained challenging [4,5]. For unresectable pancreatic cancer, treatment with chemotherapeutic agents is the mainstream option [6,7]. Sophisticated treatment options, such as those using targeted immunological therapies (vismodegib and erlotinib), have not proven successful to date [6]. Adjuvant chemotherapy alone or with radiation has been widely used in the clinical set up to improve prognosis status [8]. However, severe side effects limit the use of chemo-radiotherapy. Clearly, there is a need to evaluate new treatment modalities that can address and overcome adverse clinical manifestations.

The proximity of the liver is problematic during radiotherapy of pancreatic cancer. Significant hepatic exposure is expected and the radiosensitivity of the liver causes debilitating injury [9]. Hepatic cells, such as Kupffer cells (KC), sinusoidal endothelial cells (SEC) and hepatic stellate cells, are known to be radiosensitive. They release various substances that promote liver fibrosis, contributing to distorted liver structure and function during radiation treatment [10,11,12]. Early effects of irradiation include DNA damage, oxidative stress and production of reactive oxygen species. This leads to hepatocellular apoptosis and acute inflammatory responses [13]. Drug substances that can exert simultaneous anticancer activity and radiation protection with minimal side effects are potential candidates for optimal treatment of pancreatic malignancies.

Extracts or pure compounds from natural products (natural sources: plants, marine organisms and microorganisms) are routinely screened for their anti-tumor and radioprotective properties. Among these, there is sizeable structural diversity and chemical complexity and they modulate several cellular-signaling pathways [14,15].

Crocin (crocetin digentiobiose ester) is a unique water-soluble carotenoid isolated from the dried stigma of flowers of *Crocus sativus* L. (Saffron) and the primary component contributing to the bright red color of saffron [16]. Previous studies have reported antihyperlipidemic [17] and cardioprotective [18,19,20] effects, together with hypotensive and antidepressant properties [21,22,23]. Crocin is also known to possess active anticancer activity against several types of tumor cells [24,25]. Various studies have shown that carotenoids of saffron extract, crocin and crocetin, arrest the cell cycle at S, G0/G1and G2/M stages [26,27,28,29,30,31,32,33,34], inhibiting mitosis, cell proliferation and triggering apoptosis. Crocin is also known for its protective effects on the heart, skeletal muscles and retinal tissues during oxidative insults [35]. It has been reported to be effective in protecting a variety of organs against radiation-related injuries such as liver, kidneys, heart, skeletal muscles [35]. Crocin reduced testicular damage and exhibited significant protection against radiation-induced hydroxyl radical DNA strand breaks [36].

We have previously reported that crocin induces DNA fragmentation, apoptosis and cell cycle arrest at the G1 phase in Bx-PC-3 pancreatic cancer cells [24]. This study suggests that crocin inhibits the proliferation of BxPC3 cells through the induction of apoptosis by acting on DNA and activation of P53 signaling pathway [24]. However, the exact molecular mechanism is not elucidated yet. In this present study, we present the first report on the detailed mechanism of crocin-induced pancreatic cancer cell death. Furthermore, we demonstrate that crocin induces restoration of ionizing radiation-induced liver impairment in vivo.

## 2. Materials and Methods

### 2.1. In Vitro Thiobarbituric Acid Reacting Substances Assay

The thiobarbituric acid reacting substances (TBARS) assay was used as described previously [37]. The reaction mixture contained concentrations of crocin (5-, 10-, 25-, 50-, 100-, 150-, 200-, 250-, 300-, 400-, 500-µg mL^−1^) in a final volume of 0.5 mL. The reaction mixture was incubated at 37 °C for 1 h, then 0.4 mL removed and treated with 0.2 mL sodium dodecyl sulfate (SDS) (8.1%), 1.5 mL thiobarbituric acid (TBA) (0.8%) and 1.5 mL acetic acid (20%, pH 3.5). The total volume was made up to 4.0 mL with distilled water and kept in a water bath at 95 °C to 100 °C for 1 h. After cooling, 1.0 mL of distilled water and 5.0 mL of *n*-butanol and pyridine mixture (15:1 *v/v*) were added to the reaction mixture, shaken vigorously and centrifuged at 4000 rpm for 10 min. The butanol-pyridine layer was removed and absorbance at 532 nm measured.

### 2.2. Chemicals and Reagents

The human pancreatic cancer cell lines (Bxpc-3 and Capan-2) were purchased from ATCC, (Manassas,Virginia, USA). Dulbecco’s modified Eagle’s medium—high glucose (DMEM-H) (Cat. No. 11965092) and fetal bovine serum (FBS) (Cat. No. 16000036) were purchased from Gibco/Invitrogen (Carlsbad, CA, USA). Analytical grade dimethyl sulfoxide (DMSO), trypsin, streptomycin, penicillin, 3-[4,5-dimethyl-2-thiazolyl]-2,5- diphenyl-2-tetrazolium bromide (MTT) was purchased from Selleck (USA). Bcl-2, Bax, cleaved-caspase-3, pro-caspase-3, P38, P21^Cip1^, P27^Kip^, P53, c-Myc, CDK2 and β-actin antibodies were purchased from cell signaling technology UK.

### 2.3. Cell Lines and Culture Method

BXPC3 and Capan-2 cells were purchased from ATCC, USA. Cells were cultured in RPMI 1640 medium with 10% fetal bovine serum and 1% antibiotics (penicillin/streptomycin). Cells were maintained in a humidified cell incubator at 37 °C and 5% CO_2_ atmosphere.

### 2.4. Drug Preparation

A stock solution of crocin was prepared in dimethyl sulfoxide. Different concentrations of (10, 20, 30 and 40 µg mL^−1^) were prepared in 1% DMSO and added in the cell culture medium. Doxorubicin 5-µg/mL concentration was used as a positive control.

### 2.5. MTT Assay

Pancreatic cancer (BxPC3 and Capan-2) Cells (1 × 10^5^/well) were seeded in 96 well plates (100 μL per well) and allowed to adhere firmly overnight in RPMI medium with 10% fetal bovine serum. Then cells were treated with different concentrations (10, 20, 30 and 40 µg/mL) of crocin for 24 h. After 24 h, the medium was removed and cells incubated with MTT reagent (5 mg mL^−1^) for 4 h and violet formazan crystals dissolved in dimethyl sulfoxide and absorbance read at 540/690 nm. The absorbance of the control (without treatment) was considered as 100% cell viability. Doxorubicin was used as a positive drug control.

### 2.6. Western Blot Analysis

The whole-cell lysates prepared from BXPC3 and Capan-2 cells were treated with crocin (10, 20, 40 µg/mL) for 24 h, as described earlier [38]. The whole cell lysate was resolved in SDS (10%) poly(acrylamide) gel electrophoretically and electro-transferred onto a nitrocellulose membrane. The immunoblots were probed with Bcl-2, Bax, caspase 3 (cleaved), Caspase 9 (cleaved), P53, P21^cip1^, P27^kip1^, CDK2, c-MYC antibodies and were visualized with NBT/BCIP chromogenic substrate. Furthermore, cytochrome c release from both mitochondrial and cytosolic fractions of crocin treated BXPC3 cell lysate was collected at 12, 24 and 36 h as previously described [39] and the expression of cytochrome c release was analyzed by probing with a specific monoclonal antibody.

### 2.7. Gene Expression Profile Using Microarray Analysis

Logarithmically growing BxPc-3 pancreatic cancer cells (1 × 10^7^) were incubated for 6 h (37 °C, 5% CO_2_ atmosphere) in the presence and absence of crocin (10 µg mL^−1^). After incubation, attached cells were scraped off, collected in plastic vials, pelleted using centrifugation at 5000× *g* and stored under liquid nitrogen. Total RNA and cRNA were purified (Qiagen’s RNeasy mini kit , Manchester, UK) and RNA quality checked (Bio-analyzer). The slides used for analysis were of Agilent’s Human Array G8451B. The labeling method was T7 promoter based-linear amplification to generate labeled complementary RNA (one-color microarray-based gene expression analysis). The data analysis for gene normalization was completed by using Gene Spring GX version 7.3 and Microsoft Excel.

### 2.8. In Vivo Radiation-Induced Liver Toxicity

Swiss albino mice (*n* = 6) were used for this study. Mice were divided into four groups. Group 1: mice received distilled water subcutaneously. Group 2: Mice were injected subcutaneously. With 100 mg crocin per kg bodyweight for five consecutive days. Group 3: Mice were injected subcutaneously with PBS for five straight days and exposed to 4 Gy of gamma radiation for 30 min, 1 h, 2 h and 4 h. Group 4: Mice were injected with 100 mg crocin per kg bodyweight for five consecutive days and exposed to 4 Gy of gamma radiation for 30 min, 1 h, 2 h and 4 h. After the experimental period, the liver was perfused and homogenized. The homogenate was centrifuged at 1000 rpm for 10 min at 4 °C to obtain a supernatant. The supernatant was analyzed for hepatotoxicity markers, as described previously [40,41].

### 2.9. In Vivo Tumor Xenograft

Female athymic nude mice (NCR nu/nu) 6–8 weeks old were obtained from Vivo Bio Tech, Ltd., Hyderabad, India. The study carried out as per OECD guidelines for testing for chemicals under IAEC number JNCHRC/13/IAEC/PN-185/B. All experiments were conducted under the guidance of the Institutional Animal Ethical Committee (Project No.500/01/08/2016/project 5/28/07/2019) and PPL No. 2768 these conformed to the guidelines set by WHO (World health organizations, Geneva, Switzerland & INSA, New Delhi and Under The Animal (scientific procedures) Act 1986, respectively. All Animal work was done in UK and India by two investigators (MMT and HAB). The crocin in vivo drug doses were determined by toxicity studies (data not shown) conducted as OECD guidelines.

Mice were fed an antioxidant-free AIN-76A special diet a week before starting the trial. Establishing the tumor xenograft model in athymic nude mice was performed as described previously [42]. Briefly, BXPC-3 cells (1.0 × 10^6^) in 0.1 mL PBS were injected subcutaneously in both flanks of nude mice.

In this study, we used resource equation method for the calculation of sample size for animal studies [43,44].

Mice were divided randomly into three groups with six mice in each group. Since each mouse had two tumors, every group consisted of 12 tumors. Group 1 (controls) received 0.1 mL PBS by oral gavage. Group 2 received a daily dose of 50 mg crocin per kg body weight five times per week (Monday–Friday), Group 3 received a daily dose of 100 mg crocin per kg five times per week (Monday–Friday), respectively, in 0.1-mL PBS by oral gavage. Treatment commenced the same day after tumor cell implantation. Crocin was dissolved in PBS and filtered (0.22 μm) before administering it to the mice. Tumors were measured by Vernier scale calipers three times per week (Monday, Wednesday, Friday) and each mouse was weighed twice per week (Monday and Friday).

## 3. Histology of Liver Tissue

At the end of the experimental period, mice were euthanized by cervical dislocation and the liver dissected and sectioned. The tissue section was stained with hematoxylin and eosin, as described previously [42]. Microscopic evaluations were performed by a pathologist.

### Densitometry and Statistical Analysis

The intensity of immunoreactive bands was determined using a densitometer (Molecular Dynamics, Sunnyvale, CA, USA) equipped with Image QuaNT software. Results are expressed as mean values with 95% confidence intervals. All statistical calculations were performed using InStat software and GraphPad Prizm 4.0. Nonparametric analysis of variance (ANOVA) followed by Bonferroni post hoc multiple comparison tests were used to test the statistical significance between multiple control and treated groups. The Student’s t test was used to compare control and treated group only. Differences were considered significant at *p* < 0.05.

## 4. Results

### 4.1. Inhibition of Lipid Peroxidation by Crocin (In Vitro)

**Figure 1:**
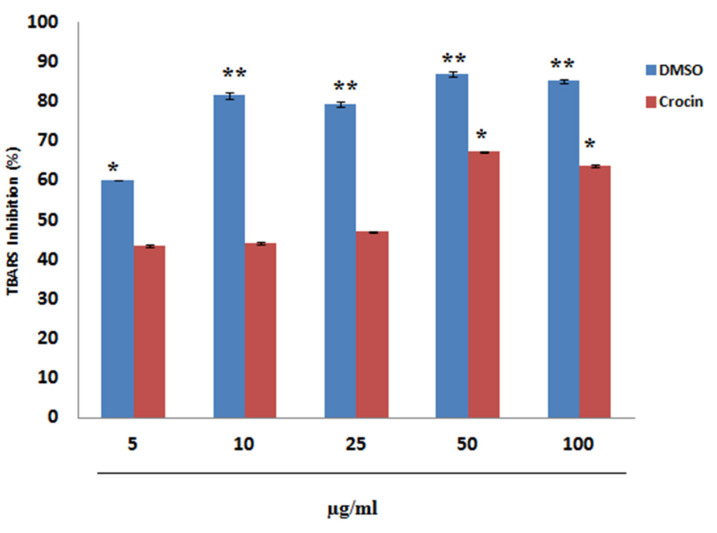
In vitro inhibition of thiobarbituric acid reacting substances (TBRS) by crocin. Data presented as mean ± SD of triplicates of three independent experiments. .*Asterisks represents significance (* *p* < 0.05; ** *p* < 0.01)

TBARS (Thiobarbituric acid reactive substances) assay is well known to mimic lipid peroxidation of the system. In this study, in vitro antioxidant ability of crocin was tested using TBARS assay. Crocin showed dose-dependent inhibition of TBARS (Figure 1) this indicates that crocin is a potent antioxidant compound.

### 4.2. Cytotoxicity of Crocin on BXPC3 Cells and Capan-2 Cells

BXPC3 cells and Capan-2 cells treated with crocin at 10, 20 and 40-µg/mL showed a significant dose-dependent decline in cell viability (Supplementary Figures S1 and S2).

### 4.3. Role of Crocin on Apoptosis in BXPC3 Cells and Capan-2 Cells

**Figure 2:**
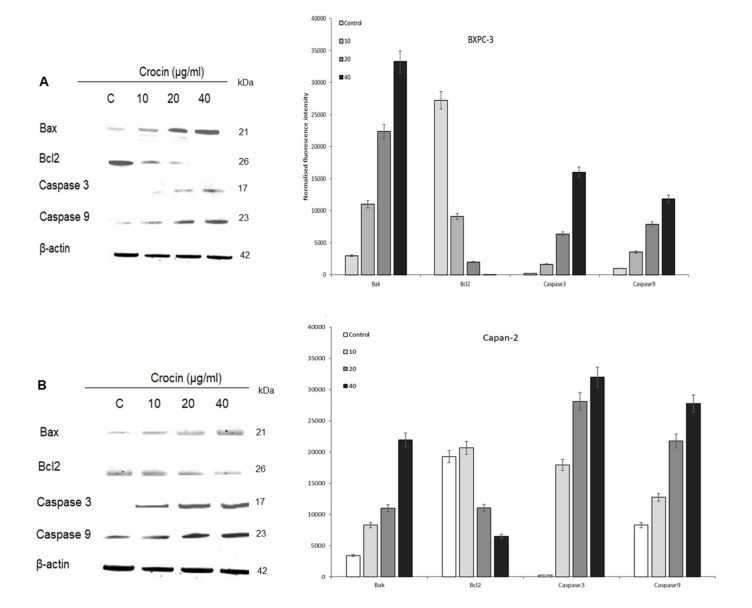
Role of crocin on BXPC3 and Capan-2 cells caspase signaling. BXPC3 and Capan-2 cells treated with different concentration (10, 20 and 40 µg/mL) of crocin for 24 h The whole-cell lysate prepared from crocin treated BXPC3 and Capan-2 cells and resolved in SDS PAGE. Analysis. Resolved proteins immune probed with BAX, Bcl2, Caspase3 and Caspase 9 antibodies. β-actin as a loading control. (**A**) Dose-dependent effect of Crocin on BXPC3 caspase signaling and protein band quantification by densitometric analysis; (**B**) dose-dependent effect of crocin on Capan-2 caspase signaling and protein band quantification by densitometric analysis with control being 100%.

Crocin induced apoptosis in BXPC3 cells and Capan-2 cells dose-dependently. Crocin upregulated BAX with simultaneous downregulation of Bcl2. Further caspase 3 and caspase 9 upregulated upon dose-dependently upon crocin treatment. The data indicate that crocin executes apoptosis in BXPC3 cells via caspase signaling (Figure 2A,B).

### 4.4. Effect of Crocin on Cytochrome C Release in BXPC3 Cells

**Figure 3:**
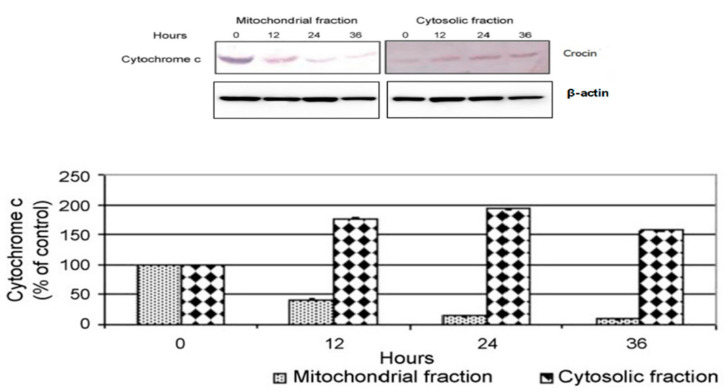
Crocin induced release of mitochondrial cytochrome c in BXPC3 cells. Release of cytochrome c from mitochondria to the cytosol was detected in BxPC-3 cells treated with crocin (10 µg/mL) at a time point of 0, 12, 24 and 36 h. The protein bands were subsequently quantified by densitometric analysis with that of control being 100% as shown just below the immunoblot data. Data represented the mean ± SEM of three independent experiments.

Crocin induced significant cell death in BXPC3 cells (25); however, the mechanism of cell death was unclear. In this study, we found crocin treatment elicits time-dependent cytochrome c release from mitochondria to cytosol in BXPC3 cells (Figure 3). Cytochrome c is released from the mitochondria to the cytosol during apoptosis when mitochondrial membrane permeability is disrupted (Figure 3).

### 4.5. P53 and Cell Cycle Proteins Expression in Crocin Treated BXPC3 Cells and Capan-2 Cells

**Figure 4:**
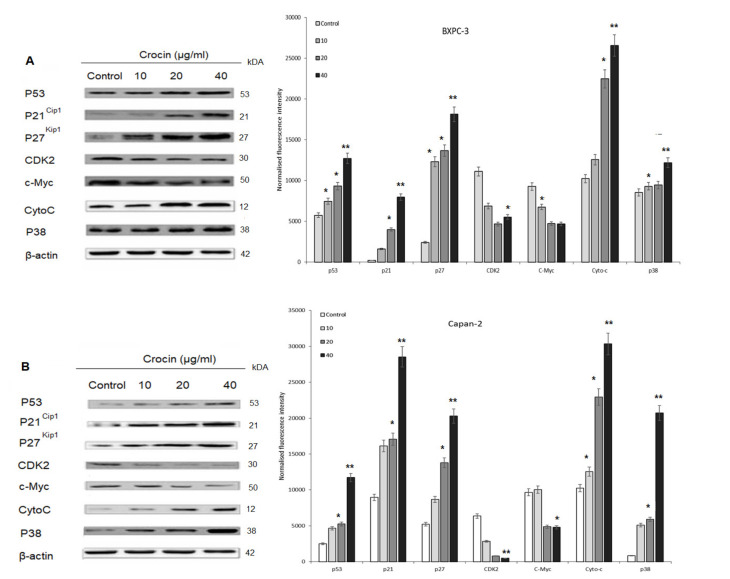
Effect of crocin on cell cycle signaling proteins in BXPC3 and Capan-2 cells. BXPC3 and Capan-2 cells treated with different concentration (10, 20 and 40 µg/mL) of crocin for 24 h The whole-cell lysate prepared from crocin treated BXPC3 and Capan-2 cells and resolved in SDS-PAGE. Resolved proteins immune probed with P53, P21Cip1, P27Kip1, CDK2, c-Myc, Cyto-C and P38 antibodies. β-actin as loading control; (**A**) Dose-dependent effect of Crocin on BXPC3 cell cycle signaling and protein band quantification by densitometric analysis; (**B**) dose-dependent effect of crocin on Capan-2 cell cycle signaling and protein band quantification by densitometric analysis with control being 100%. Data represent mean ± SEM. * indicates significant differences compared to control (* *p*0.05; ** *p*0.01).

Crocin treated BXPC3 cells and Capan-2 cells were analyzed for expression of different cell cycle proteins such as P21^cip1^, P27^kip1^, CDK2 and CMYC, including P53. Crocin upregulated P53, P38, cytochrome c, P21^cip1^ and P27^kip1^, whereas CDK2 and c-Myc were downregulated dose-dependently in BXPC3 and Capan-2 cells (Figure 4A,B).

### 4.6. Identification of Crocin Treated BXPC3 Cells Gene Signature

**Figure 5:**
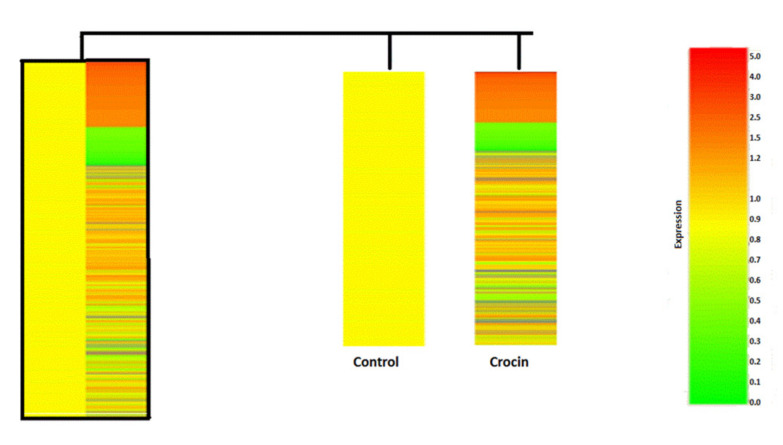
Crocin induced gene signatures of BXPC3 cells. BxPC3 cells (1 × 10^7^) were treated with crocin (10 µg/mL). Total RNA was isolated from crocin treated BXPC3 cells and hybridized with Agilent’s Human Array G8451B.

Crocin treated BXPC3 cells demonstrated changes in expression patterns of genes involved in several pathways and checkpoints (Figure 5). Microarray analysis showed that crocin treatment altered the expression of a total of 723 genes, including 269 upregulated and 454 down regulated (see Supplementary Materials). Among the upregulated genes due to crocin treatment involved in the regulation of several checkpoints and pathways (Tables S1 and S2).

### 4.7. In Vivo Pancreatic Tumor Remission by Crocin

**Figure 6:**
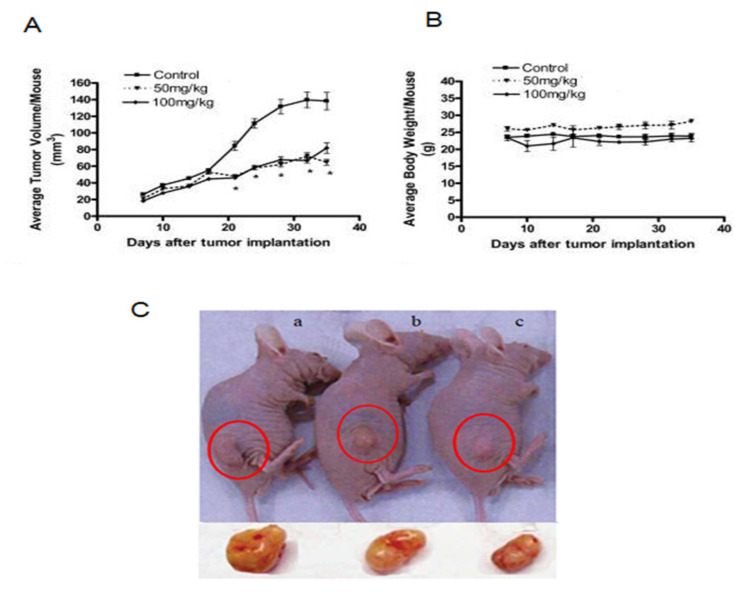
Efficacy of crocin on in vivo pancreatic tumor remission and bodyweight of tumor-bearing mice. (**A**) Tumor remission by 50 mg/kg and 100 mg/kg of crocin treatment; (**B**) body weight of tumor-bearing mice; (**C**) tumor remission; (**a**) control; (**b**) 50 mg/kg treatment of crocin; (**c**) 100 mg/kg treatment of crocin.

As can be seen in Figure 6A, for the first 18 days, no difference was observed for treated (50 and 100 mg/kg body weight) and non-treated mice. However, at the end of the treatment, an average of approximately 52% drop in tumor volume is observed between both treatment regimens and non-treated mice. Indeed, no significant difference is found between the two dosage regimens evaluated. Furthermore, no significant changes in body weight are observed during the 40 days treatment; Figure 6B.

### 4.8. Restoration of Radiation Induced Liver Toxicity by Crocin (In Vivo)

**Figure 7:**
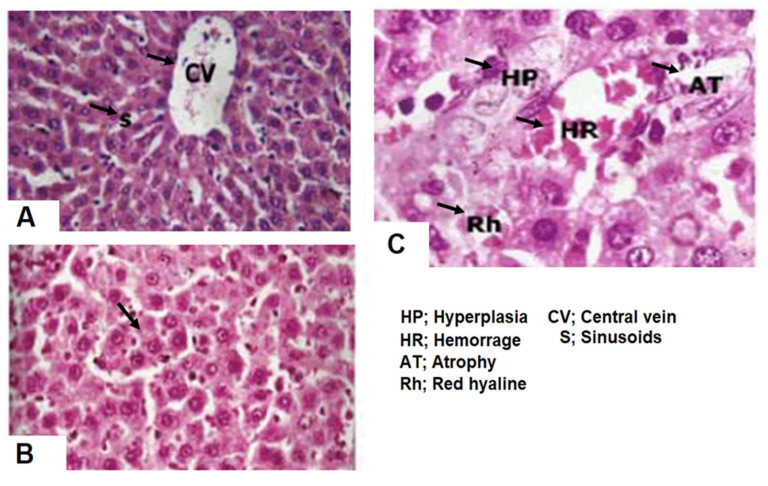
Restoration of radiation-induced liver damage by crocin. Photomicrographs of liver tissue. (**A**) Control groups: Normal histological appearance of liver tissue; (**B**) PBS + 4Gy-treated groups: hyperplasia (arrow), hemorrhage, atrophy and red hyaline (arrowhead); (**C**) crocin (100 mg/kg, but) + 4Gy-treated groups: regular hepatocytes in the most region and mild activated Kupffer cells. H&E Staining, original magnifications: ×200.

Crocin (100 mg/kg) pre-treatment reversed the damage caused by radiation by maintaining structural integrity of hepatocytes and number of Kupffer cells (Figure 7). Changes in biochemical parameters such as reduced glutathione (GSH), glutathione peroxidase (GPx), glutathione reductase (GR), glutathione transferase (GT) and malondialdehyde (MDA) are clinical manifestation of system toxicities including radiation-induced hepatotoxicity. In this study, levels of GSH (30 min: 16.6 ± 0.11; 1 h: 13.3 ± 0.08; 2 h: 6.6 ± 0.05; 4 h: 3.33 ± 0.03) GPx (30 min: 2.4 ± 0.1; 1 h: 2.3 ± 0.02; 2 h: 2.0 ± 0.12; 4 h: 1.8 ± 0.05), GR (30 min: 0.65 ± 0.02; 1 h: 0.5 ± 0.003; 2 h: 0.4 ± 0.008; 4 h: 0.15 ± 0.02), GT (30 min: 1.75 ± 0.02; 1 h: 1.04 ± 0.02; 2 h: 1.02 ± 0.01; 4 h: 1.0 ± 0.006) and MDA (30 min: 0.1 ± 0.006; 1 h: 0.2 ± 0.005; 2 h: 0.2 ± 0.01; 4 h: 0.3 ± 0.01) were altered time dependently in mice received 4 Gy of irradiation. Pretreatment with crocin (100 mg/kg body weight) reversed the changes caused by irradiation and brought back the levels of biochemical parameters to near normal levels (Table 1 and Table 2).

## 5. Results

Ionizing radiation treatment regimens have been modernized to precisely target the tumor site. However, the detrimental effects of radiation on the organs anatomically situated near the tumor site is currently inevitable. Pancreatic cancer patients who receive radiotherapy as a part of treatment protocols sensitize their liver leading to hepatotoxicity. For improved therapeutic outcomes, an ideal drug would be one that offers pancreatic anticancer efficacy as well as protection against radiosensitization. Here, we provide evidence for the mechanism of cell cycle arrest by crocin and demonstrate the potential for protection against radiosensitization by this carotenoid extracted from saffron.

Apoptosis occurs primarily via two pathways: the mitochondrial or through death receptor pathway. Bcl-2 is involved in cell survival and inhibits cell apoptosis induced by a variety of stimuli, indicating that Bcl-2 is a negative regulator of cell apoptosis [45,46]. Bax is a pro-apoptotic protein, which resides in the outer mitochondrial membrane and translocates to the mitochondria at an early stage of apoptosis. Therefore, a reduced Bcl-2 expression accompanied by high expression of Bax may promote the apoptotic response to anticancer drugs [47]. In this study, we found that in BXPC3 and Capan-2 cells, crocin reduces the Bcl-2 expression and upregulates Bax expression in a dose-dependent manner (Figure 2). This alteration in the Bcl-2/Bax ratio indicates the apoptosis-inducing potential of crocin in BXPC3 and Capan-2 cells. This is in agreement with previous results [26,46,47,48], in which crocin is shown to affect the Bax/Bcl2 ratio to cause apoptosis in Leukemia, lung cancer, AGS gastric cancer and prostate cancer cells.

Numerous studies have shown that active caspase-3 is needed to induce apoptosis in response to chemotherapeutic treatments [49,50,51,52]. To dissect the molecular mechanism of caspase-mediated apoptosis by crocin in BXPC3 and Capan-2 cells, we studied the expression level of active caspase 3, caspase 9 and cytochrome c. Our results indicate that crocin markedly upregulates the expression of active caspase 3, 9 and cytochrome c in BXPC3 and Capan-2 cells, respectively (Figure 2 and Figure 4). It further elicits time-dependent upregulation of cytosolic cytochrome c in BXPC3 cells (Figure 3). Crocin induced hallmark of apoptosis in BXPC3 cells and Capan-2 cells by cleaving the pro-apoptotic caspase 3. Cleavage of caspase 3 is the sign of post-translational modification, which leads to the formation of the death-inducing signaling complex. In addition, the release of cytochrome C from the inner membrane of mitochondria is essential for the initiation of the apoptotic cascade [53,54]. In this study, crocin facilitated the release of cytochrome c from mitochondria in BXPC3 cells, which evidenced that crocin mediated apoptosis in BXPC3 cells via caspase and mitochondrial-dependent pathway.

P53 is the tumor suppressor and one of the most frequently mutated genes in 70% of pancreatic cancers. P53 transcriptionally activates the genes in response to acute DNA damage-inducing proliferative arrest or apoptosis [55]. Clinical data suggest that poor prognosis status of pancreatic ductal adenocarcinoma patients associated with low P53mRNA transcript level [56]; thus, P53 expression can be used as a biomarker for prognosis and therapy prediction. In this study, we found that crocin treatment upregulates the P53 expression in BXPC3 and Capan-2 cells (Figure 4).

Following our results, crocin induced apoptosis by upregulating the P53 expression in cervical, brain and breast cancer cells [57,58,59]. However, the molecular mechanism of interaction of crocin and P53 is not well understood. DNA damage regularly initiates the p53 pathway resulting in cell cycle arrest at the G1 phase to restore genome stability and avoid subsequent malignancy. Cyclin-dependent kinase (CDK) complexes are formed and activated at specific stages of the cell cycle and their activities required for progression through distinct cell cycle phases [60]. The dysregulation of CDK in the cell cycle is often found elevated in human tumors and is associated with the unrestrained proliferation of cells, an essential hallmark of cancer [61]. Aberrant activation of CDKs which seen in human diseases, provided a rationale for designing inhibitors of CDKs as anticancer drugs [62].

It is known that p53 regulates cell cycle by suppressing CDK’s complex activation and by facilitating CDK’s binding with endogenous inhibitors proteins p21/CIP1 and p27/KIP1, which results in inhibition of CDK’s kinase activities and prevents cell cycle progression. In this study, crocin treatment reduced the expression of CDK2 and upregulated the expression of p21/CIP1 and p27/KIP1 in BXPC3 cells and Capan-2 cells, respectively. Current data agreed with our previous report, where crocin cease the cell cycle at the G1 phase in BXPC3 cells. It is well established that the G1 phase of the cell cycle-regulated by CDK2 complex [63] and its inhibition can prevent the cells from getting through the G1 phase and entering S-phase of the cell cycle.

Further up-regulation of p21/CIP1 and p27/KIP1 by crocin treatment indicates that crocin enhances binding of p21/CIP1 and p27/KIP1 with CDK2; thus, CDK2 lost its kinase activity and prevented the progression of cells through the G1/S transition. In addition to this, several studies provide evidence that activation of p38 resulted in inhibition of the cell cycle at the G1/S transition [64]. p38 can modulate the retinoblastoma protein (pRb) phosphorylation, which is a crucial regulator of G1 checkpoint; thus, p38 can orchestrate cell cycle arrest through CDK’s independent way. Activation of p38 depends on the p53 expression because p53 serves as an in vitro and in vivo substrate for p38 in different models [65,66]. Our study shows that crocin treatment profoundly enhances the expression of both p53 and p38 in BXPC3 and Capan-2 cells dose-dependently (Figure 4). Crocin could play a functional role in enhancing the binding of p53 and p38 and this interaction could halt the progression of the cell cycle in BXPC3 and Capan-2 cells. Transcriptional activation of cell cycle genes by c-MYC is essential to regulate cell proliferation, cell differentiation and apoptosis [67]. c-MYC is a critical transcriptional factor and its overexpression promotes the progression of the cell cycle through the G1/S transition. Previous studies reported that in transgenic mouse models have an insight that transient inactivation of the c-MYC leads to tumor regression. Thus, suggesting that the regulation of oncogenic c-MYC harnessed to treat cancer patients [68]. Recent studies enumerated that c-MYC represents a central oncogene in pancreatic cancer and its expression correlated with the perineural invasion and poor prognostic features [69]. Therefore, we studied the role of crocin on expression c-Myc expression in BXPC3 and Capan-2 cells. We found that crocin treatment abrogates the c-Myc expression in BXPC3 and capan-2 cells dose-dependently (Figure 4). C-MYC predominantly located in the nucleus; therefore, pharmacological inhibition of c-MYC requires localization of drugs at the nuclear site. Our data suggest that crocin could localize into the nucleus of BXPC3 and capan-2 cells and suppress the Myc expression. Consistent with this notion in the present study, we demonstrate that increased p53, p38, p21/p27 expression and downregulation of CDK2 and c-MYC levels could be an explanation by which BXPC3 and Capan-2 cells undergo cell cycle arrest during crocin exposure. Crocin could target multiple signaling molecules simultaneously to arrest BXPC3 and Capan-2 cell growth. However, additional experiments such as pharmacological inhibition of critical molecules and drug localization upon crocin treatment in BXPC3 and Capan-2 cells will be required to understand the precise molecular mechanism of crocin inducing cell cycle arrest.

The occurrence and progression of pancreatic cancer are associated with differential expression of genes. Cancer genomic studies have advanced our understanding of the pathogenesis of pancreatic cancer significantly. Identifying drug targeting genes and its downstream in the tumor would be a tremendous effort which would facilitate the use of a drug in patients who require precision medicine. In this study, we performed microarray analysis to acquire an overall view of crocin inducing signaling pathways in BXPC3 cells. Our data reveals that a total of 723 genes, including 269 upregulated and 454 down regulated, in crocin treated BXPC3 cells. In the present study, crocin up-regulated genes involved in different checkpoints such as cell cycle, DNA damage, DNA damage, mitotic spindle, programmed cell death, regulation of cell differentiation, cell death and cell adhesion (Table S1). This observation supports the notion of crocin as a pro-apoptotic and cell cycle arresting agent. Crocin up-regulates GADD45β is a vital gene activated during p53 dependent pro-apoptotic pathway and it could also enable the caspase cascade leading the cell to apoptosis in BXPC3 cells. Prostaglandin (PG) is a bioactive lipid that impacts healthy development, tissue homeostasis, inflammation and cancer progression [70]. The inflammatory and carcinogenic activity increased the expression of COX-1 and microsomal PGE synthase-1 (mPGES-1). Both occur to amplify the accumulation of PGE2 in tumors [71]. In this study, PG inducible genes such as prostaglandin E synthase (PTGES), prostaglandin–endoperoxidase synthase 1 (PTGS1), COX1, phospholipase A2- group 5 (Pla2g5), cytochrome P450, family 2, subfamily c, polypeptide 37 (Cyp2c37) were downregulated by crocin treatment (Table S2). COX is the key enzyme required for the conversion of arachidonic acid to prostaglandins [72]. Arachidonic acid metabolized by cytochrome *P-*450 type enzyme systems to produce hydroxy and carboxy products [73]. It indicates crocin could ameliorate pancreatic tumor progression by suppressing arachidonic acid metabolism. Furthermore, downregulated genes were carbonyl reductase 2 (Cbr2) and oncogenes Ras-related GTP binding D.

The MAP kinase kinase kinase 6 (MAPKKK6), inflammatory genes bradykinin receptor, beta 1 (Bdkrb1), nitric oxide synthase 3 (NOS 3), fibroblast growth factor (FGF), hypoxia-inducible factor 3 (HIF3) were present, among others. The significance of this crocin mediated downregulation of these genes remains unclear. Further specific gene knocks out are warranted to unravel the mechanism of action of crocin.

Crocin found to induce cell death in BXPC3 and Capan-2 cells by triggering caspase signaling; however, safety and tumor efficacy of the crocin in vivo pancreatic cancer model is unclear. To address this issue, in this study, we develop an in vivo pancreatic tumor model and assess the tumor remission property of crocin. Crocin treatment significantly reduce tumor burden and no change in the bodyweight of tumor-bearing mice was observed (Figure 6). Our data indicate that crocin is a profound anti pancreatic cancer agent with less toxicity. Pancreatic cancer patients receive a cycle of ionizing radiation (IR) treatment to suppress the tumor growth unavoidably, exposing organs situated near the pancreas to IR. Anatomically liver located near to pancreas; therefore, radiation-induced hepatic damage is visible. IR induced oxidative stress is a known pathologic consequence is due to aberrant accumulation of reactive oxygen species (ROS), which can initiate liver injury. The imbalance between ROS generation and antioxidant system cause severe damage to the liver. Results show that crocin can induce BXPC3 and Capan-2 cell death by triggering cell cycle and apoptotic signaling molecules and crocin known to be a capable antioxidative molecule. In this study, we delineate the role of crocin on in vivo IR induced oxidative stress in the liver. This study adds the value on crocin not only as an anticancer molecule, but also radiation protection agent.

Crocin administration prevented liver histopathologic changes induced by IR, as evidenced by H&E staining (Figure 7). Our in vitro antioxidant data indicates that crocin can inhibit TBARS dose-dependently (Figure 1). Further, we studied the anti-lipid peroxidative role of crocin in mice. A single dose of IR (6 Gy) significantly elevated the levels of malondialdehyde (MDA). The main product of lipid peroxidation and cytotoxic nitric oxide in the liver [74,75]. In our study, we found that IR (4Gy) increased the MDA levels in mice and crocin treatment restores the MDA level (Table 2). Evidenced that crocin can protect the liver IR induced oxidative damage. IR mediated alteration of redox potential due to free radicals’ generation can cause a dramatic fall in the hepatic glutathione (GSH). Enzymes such as glutathione peroxidase, glutathione reductase and glutathione transferase lead to membrane lipid peroxidation [76,77]. Therefore, searching for an agent can reduce radiation toxicity in healthy tissues is of scientifically much interest. In this study, increased GSH level (Table 1) suggests that protection by the crocin mediated through the restoration of IR altered hepatic antioxidant status (Table 1 and Table 2). Crocin treatment confers hepatic protection by scavenging IR generated free radicals. During radiolysis and induce cellular radioprotectors such as GSH.

## 6. Conclusions

In conclusion, crocin induced pancreatic cancer cell death via cleaving caspase 3 and mitochondrial c release. Crocin downregulated expression of p53 in BXPC3 and capan-2 cells and modulated the expression of cell cycle proteins such as CDK2, P21 and P27. Microarray data reveals that crocin upregulated genes involved in different checkpoints (cell cycle and DNA damage) and downregulated genes involved in arachidonic acid metabolism. Toxicity study shows that crocin is safe and crocin reduced in vivo tumor growth. Crocin offers hepatic protection by scavenging radiation-induced alteration of antioxidant status in mice. The results from our study suggested that crocin is radioprotective as well as inducing apoptosis in pancreatic cancer which indicates its potential as clinical translation. However, before reaching a definite conclusion, the more detailed study, including a human clinical trial, is required to fully understand the role of dietary crocin in the management and treatment of pancreatic cancer.

## Figures and Tables

**Table 1:** Effect of dietary crocin on radiation-induced glutathione depletion, glutathione reductase, glutathione peroxidase activity in irradiated mice liver. Data presented as mean ± SD of triplicates of three independent experiments. ^a^: *p* < 0.0001 ^b^: *p* < 0.1 ^c^: *p* < 0.01 compared to PBS. ^1^: *p* <0.0001 ^2^: *p* < 0.1 ^3^: *p* < 0.01 compared to RT alone.

GSH (µM/mg Tissue)	30 min	1 h	2 h	4 h
PBS	56.6 ± 0.05	50 ± 0.20	46.6 ± 0.05	50 ± 0.18
Crocin (100 mg/kg bwt)	63.6 ± 0.03 ^a^	56.6 ± 0.1 ^a^	56.6 ± 0.1 ^a^	53.3 ± 0.20 ^a^
PBS + RT (4 Gy.)	16.6 ± 0.11	13.3 ± 0.08	6.6 ± 0.05	3.33 ± 0.03
Crocin (100 mg/kg bwt) + RT(4Gy)	63.3 ± 0.08 ^1^	60 ± 0.18 ^1^	56.6 ± 0.1 ^1^	46.6 ± 0.05 ^1^
GPx (µM/min/mg/protein)				
PBS	2.4 ± 0.05	2.5 ± 0.12	2.8 ± 0.05	2.83 ± 0.06
Crocin (100 mg/kg bwt)	2.8 ± 0.05 ^c^	2.9 ± 0.05 ^b^	2.9 ± 0.1	3.06 ± 0.12
PBS + RT (4 Gy.)	2.4 ± 0.1	2.3 ± 0.02	2.0 ± 0.12	1.8 ± 0.05
Crocin (100 mg/kg bwt) + RT(4Gy)	2.7 ± 0.11	2.6 ± 0.11 ^2^	2.5 ± 0.05 ^2^	2.33 ± 0.08 ^3^
GR(µM/min./mg/protein)				
PBS	0.3 ± 0.05	0.25 ± 0.05	0.2 ± 0.006	0.25 ± 0.05
Crocin (100 mg/kg bwt)	0.3 ± 0.005	0.4 ± 0.02 ^b^	0.4 ± 0.01 ^a^	0.5 ± 0.0066 ^b^
PBS + RT (4 Gy.)	0.65 ± 0.02	0.5 ± 0.003	0.4 ± 0.008	0.15 ± 0.02
Crocin (100 mg/kg bwt) + RT(4Gy)	1.1 ± 0.04 ^1^	0.9 ± 0.04 ^1^	0.7 ± 0.01 ^1^	0.6 ± 0.01 ^1^

**Table 2:** Effect of dietary crocin on radiation-induced glutathione transferase activity and rate of lipid peroxidation in irradiated mice liver. Data presented as mean ± SD of triplicates of three independent experiments. ^b^: *p* < 0.1 ^c^: *p* < 0.01 compared to PBS. ^1^: *p* <0.0001 ^2^: *p* < 0.1 ^3^: *p* < 0.01 compared to RT alone.

GT(µM/min/mg/Protein)	30 min	1h	2 h	4 h
PBS	1.4 ± 0.05	1.2 ± 0.05	1.3 ± 0.05	1.1 ± 0.1
Crocin (100 mg/kg bwt)	1.5 ± 0.05	1.5 ± 0.11^b^	1.3 ± 0.05	1.4 ± 0.11
PBS + RT (4Gy.)	1.75 ± 0.02	1.04 ± 0.02	1.02 ± 0.01	1.0 ± 0.006
Crocin (100 mg/kg bwt) *+* RT (4 GY)	1.9 ± 0.05 ^2^	1.6 ± 0.11^3^	1.4 ± 0.05 ^3^	1.2 ± 0.11
MDA (µg/mg protein)				
PBS	0.05 ± 0.003	0.1 ± 0.008	0.1 ± 0.01	0.03 ± 0.003
Crocin (100 mg/kg bwt)	0.02 ± 0.01 ^c^	0.03 ± 0.003 ^c^	0.1 ± 0.008	0.02 ± 0.001 ^b^
PBS + RT (4 Gy.)	0.1 ± 0.006	0.2 ± 0.005	0.2 ± 0.01	0.3 ± 0.01
Crocin (100 mg/kg bwt) + RT(4Gy)	0.1 ± 0.01	0.1 ± 0.006 ^1^	0.1 ± 0.003 ^2^	0.15 ± 0.015 ^1^

## Supplementary Materials

The following are available online at https://www.mdpi.com/2072-6643/12/6/1901/s1, Figure S1. Cytotoxicity of crocin on BXPC3 cells. BXPC3 cells were treated with 10, 20, 30 and 40 µg/mL of crocin, Figure S2. Cytotoxicity of crocin on Capan-2 cells. Capan-2 cells were treated with 10, 20, 30 and 40 µg/mL of crocin, Table S1. Crocin enriched checkpoints and pathways of BXPC3 cells, Table S2. Crocin downregulated checkpoints and pathways in BXPC3 cells.

## References

[1] Rahib L., Smith B.D., Aizenberg R., Rosenzweig A.B., Fleshman J.M., Matrisian L.M. (2014). Projecting cancer incidence and deaths to 2030: The unexpected burden of thyroid, liver, and pancreas cancers in the United States. Cancer Res..

[2] National Cancer Institute. M. (2012). SEER Cancer Statistics Factsheets.

[3] Kamisawa T., Wood L.D., Itoi T., Takamori K. (2016). Pancreatic cancer. Lancet.

[4] Sohal D.P.S., Kennedy E.B., Khorana A., Copur M.S., Crane C.H., Garrido-Laguna I., Krishnamurthi S., Moravek C., O’Reilly E.M., Philip P.A. (2018). Metastatic Pancreatic Cancer: ASCO Clinical Practice Guideline Update. J. Clin. Oncol..

[5] Groot V.P., Gemenetzis G., Blair A.B., Rivero-Soto R.J., Yu J., Javed A.A., Burkhart R.A., Rinkes I.H.M.B., Molenaar I.Q., Cameron J.L. (2019). Defining and Predicting Early Recurrence in 957 Patients With Resected Pancreatic Ductal Adenocarcinoma. Ann. Surg..

[6] Ghosn M., Ibrahim T., Assi T., Rassy E.E., Kourie H.R., Kattan J. (2016). Dilemma of first-line regimens in metastatic pancreatic adenocarcinoma. World J. Gastroenterol..

[7] Hajatdoost L., Sedaghat K., Walker E.J., Thomas J., Kosari S. (2018). Chemotherapy in Pancreatic Cancer: A Systematic Review. Medicina.

[8] Shimoda M., Kubota K., Shimizu T., Katoh M. (2015). Randomized clinical trial of adjuvant chemotherapy with S-1 versus gemcitabine after pancreatic cancer resection. Br. J. Surg..

[9] Lewin K., Millis R.R. (1973). Human radiation hepatitis A morphologic study with emphasis on the late change. Arch. Pathol..

[10] Christiansen H., Saile B., Neubauer-Saile K., Tippelt S., Rave-Fränk M., Hermann R.M., Dudas J., Hess C.F., Schmidberger H., Ramadori G. (2004). Irradiation leads to susceptibility of hepatocytes to TNF-alpha mediated apoptosis. Radiother. Oncol..

[11] Du S.S., Qiang M., Zeng Z.C., Zhou J., Tan Y.S., Zhang Z.Y., Zeng H.Y., Liu Z.S. (2010). Radiation-induced liver fibrosis is mitigated by gene therapy inhibiting transforming growth factor-β signaling in the rat. Int. J. Radiat. Oncol. Biol. Phys..

[12] Yamanouchi K., Zhou H., Roy-Chowdhury N., Macaluso F., Liu L., Yamamoto T., Liping L., Rao G.V., Charles E., Solberg T.D. (2009). Hepatic irradiation augments engraftment of donor cells following hepatocyte transplantation. Hepatology..

[13] Robbins M.E., Zhao W. (2004). Chronic oxidative stress and radiation-induced late normal tissue injury: A review. Int. J. Radiat. Biol..

[14] Argyropoulou A., Aligiannis N., Trougakos I.P., Skaltsounis A.-L. (2013). Natural compounds with anti-ageing activity. Nat. Prod. Rep..

[15] Hakkim F.L., Miura M., Matsuda N., Alharrasi A.S., Guillemin G., Yamauchi M., Arivazhagan G., Song H. (2014). An in vitro evidence for caffeic acid, rosmarinic acid and trans-cinnamic acid as a skin protectant against γ-radiation. Int. J. Low Radiat..

[16] Hosseinzadeh H., Sadeghnia H.R., Ziaee T., Danaee A. (2005). Protective effect of aqueous saffron extract (Crocus sativus L.) and crocin, its active constituent, on renal ischemia-reperfusion-induced oxidative damage in rats. J. Pharm. Pharm. Sci..

[17] Tamaddonfard E., Farshid A.-A., Eghdami K., Samadi F., Erfanparast A. (2013). Comparison of the effects of crocin, safranal and diclofenac on local inflammation and inflammatory pain responses induced by carrageenan in rats. Pharmacol. Rep..

[18] Imenshahidi M., Hosseinzadeh H., Javadpour Y. (2010). Hypotensive effect of aqueous saffron extract (*Crocus sativus* L.) and its constituents, safranal and crocin, in normotensive and hypertensive rats. Phytother. Res..

[19] Razavi M., Hosseinzadeh H., Abnous K., Motamedshariaty V.S., Imenshahidi M. (2013). Crocin restores hypotensive effect of subchronic administration of diazinon in rats. Iran. J. Basic Med. Sci..

[20] Bakshi H.A., Hakkim F.L., Sam S., Javid F., Rashan L. (2018). Dietary crocin reverses melanoma metastasis. J. Biomed. Res..

[21] Bakshi H.A., Hakkim F.L., Sam S., Javid F. (2017). Role of Dietary Crocin in In Vivo Melanoma Tumor Remission. Asian Pac. J. Cancer Prev..

[22] Bakshi H.A., Touseef T., Fassal G. (2012). Crocus sativus L. prevents progression of cell growth and enhances cell toxicity in human breast cancer and lung cancer cell lines. Int. J. Pharma Life Sci..

[23] Bakshi H.A., Hakkim F.L., Sam S. (2016). Molecular Mechanism of Dietary Crocin Induced Caspase Mediated MCF-7 Cell Death: In Vivo Toxicity Profiling and Ex Vivo Macrophage Activation. Asian Pac. J. Cancer Prev..

[24] Bakshi H., Sam S., Rozati R., Sultan P., Islam T., Rathore B., Lone Z., Sharma M., Triphati J., Saxena R.C. (2010). DNA fragmentation and cell cycle arrest: A hallmark of apoptosis induced by crocin from kashmiri saffron in a human pancreatic cancer cell line. Asian Pac. J. Cancer Prev..

[25] Bakshi H.A., Sam S., Feroz A., Ravesh Z., Shah G.A., Sharma M. (2009). Crocin from Kashmiri saffron (Crocus sativus) induces in vitro and in vivo xenograft growth inhibition of Dalton’s lymphoma (DLA) in mice. Asian Pac. J. Cancer Prev..

[26] Hoshyar R., Bathaie S.Z., Sadeghizadeh M. (2013). Crocin triggers the apoptosisthrough increasing the Bax/Bcl-2 ratio and caspase activation in human gastricadenocarcinoma: AGS, cells. DNA Cell Biol..

[27] Yang J.Y., Della-Fera M.A., Baile C.A. (2006). Esculetin induces mitochondria-mediated apoptosis in 3T3-L1 adipocytes. Apoptosis.

[28] Hsu C.L., Yen G.C. (2007). Effects of capsaicin on induction of apoptosis and inhibition of adipogenesis in 3T3-L1 cells. J. Agric. Food Chem..

[29] Slee E.A., Harte M.T., Kluck R.M., Wolf B., Casiano C.A., Newmeyer D.D., Wang H.G., Reed J.C., Nicholson D.W., Alnemri E.S. (1999). Ordering the cytochrome c-initiated caspase cascade: Hierarchical activation of caspases 2, 3, 6, 7, 8, and 10 in a caspase-9-dependent manner. J. Cell Biol..

[30] Keane M.M., Ettenberg S.A., Nau M.M., Russell E.K., Lipkowitz S. (1999). Chemotherapy augments TRAIL-induced apoptosis in breast cell lines. Cancer Res..

[31] Bellarosa D., Ciucci A., Bullo A., Nardelli F., Manzini S., Maggi C.A., Goso C. (2001). Apoptotic events in a human ovarian cancer cell line exposed to anthracyclines. J. Pharmacol. Exp. Ther..

[32] Kottke T.J., Blajeski A.L., Martins L.M., Mesner P.W., Davidson N.E., Earnshaw W.C., Armstrong D.K., Kaufmann S.H. (1999). Comparison of paclitaxel-, 5-fluoro-2′-deoxyuridine-, and epidermal growth factor (EGF)-induced apoptosis. Evidence for EGF-induced anoikis. J. Biol. Chem..

[33] Shamas-Din A., Brahmbhatt H., Leber B., Andrews D.W. (2011). BH3-only proteins: Orchestrators of apoptosis. Biochim. Biophys. Acta..

[34] Zhong Y.-J., Shi F., Zheng X.-L., Wang Q., Yang L., Sun H., He F., Zhang L., Lin Y., Qin Y. (2011). Crocetin induces cytotoxicity and enhances vincristine induced cancer cell death via p53-dependent and -independent mechanisms. Acta Pharmacol. Sin..

[35] Samarghandian S., Farkhondeh T., Samini F., Borji A. (2016). Protective Effects of Carvacrol against Oxidative Stress Induced by ChronicStress in Rat’s Brain, Liver, and Kidney. Biochem. Res. Int..

[36] Koul A., Abraham S.K. (2018). Efficacy of crocin and safranal as protective agents against genotoxic stressinduced by gamma radiation, urethane and procarbazine in mice. Hum. Exp. Toxicol..

[37] Ohkawa H., Onishi N., Yagi K. (1979). Assay of lipid peroxidation in animal tissue by thiobarbituric acid reaction. Anal. Biochem..

[38] Habig W.H., Pabst M.J., Jakoby W.B. (1974). Glutathione S-transferases. The first enzymatic step in mercapturic acid formation. J. Biol. Chem..

[39] Revathi S., Hakkim F.L., Kumar N.R., Bakshi H.A., Rashan L., Al-Buloshi M., Hasson S.S.A.A., Krishnan M., Javid F., Nagarajan K. (2018). Induction of HT-29 Colon Cancer Cells Apoptosis by Pyrogallol with Growth Inhibiting Efficacy Against Drug-Resistant *Helicobacter pylori*. Anticancer Agents Med Chem..

[40] Tung B.T., Rodríguez-Bies E., Ballesteros-Simarro M., Motilva V., Navas P., López-Lluch G. (2014). Modulation of endogenous antioxidant activity by resveratrol and exercise in mouse liver is age dependent. J. Gerontol. A Biol. Sci. Med. Sci..

[41] Swinney D.C., Anthony J. (2011). How were new medicines discovered?. Nat. Rev. Drug Discov..

[42] Bajbouj K., Schulze-Luehrmann J., Diermeier S., Amin A., Schneider-Stock R. (2012). The anticancer effect of saffron in two p53 isogenic colorectal cancer celllines. BMC Complement. Altern. Med..

[43] Festing M.F., Altman D.G. (2002). Guidelines for the design and statistical analysis of experiments using laboratory animals. ILAR J..

[44] Festing M.F. (2006). Design and statistical methods in studies using animal models of development. ILAR J..

[45] Zhang L., Yuan X., Wang S., Ou Y., Zheng X., Wang Q. (2014). The relationship between mitochondrial Fusion/fission and apoptosis in the process of adipose-derived stromal cells differentiation into astrocytes. Neurosci. Lett..

[46] Ola M.S., Nawaz M., Ahsan H. (2011). Role of Bcl-2 family proteins and caspases in the regulation of apoptosis. Mol. Cell Biochem..

[47] Narita M., Shimizu S., Ito T., Chittenden T., Lutz R.J., Matsuda H., Tsujimoto Y. (1998). Bax interacts with the permeability transition pore to induce permeability transition and cytochrome c release in isolated mitochondria. Proc. Natl. Acad. Sci. USA.

[48] D’Alessandro A.M., Mancini A., Lizzi A.R., De-Simone A., Marroccella C.E., Gravina G.L., Tatone C., Festuccia C. (2013). Crocus sativus stigma extract and its major constituent crocin possess significant anti-proliferative properties against human prostate cancer. Nutr. Cancer.

[49] Chen S., Zhao S., Wang X., Zhang L., Jiang E., Gu Y., Shangguan A.J., Zhao H., Lv T., Yu Z. (2015). Crocin inhibits cell proliferation and enhances cisplatin andpemetrexed chemosensitivity in lung cancer cells. Transl. Lung Cancer Res..

[50] Sun Y., Wang Z., Xu H.J., Zhao Y.X., Wang L.Z., Sun L.R., Sun X.F. (2013). Crocinexhibits antitumor effects on human leukemia HL-60Cells in vitro and In vivo.Evid.-Based Compl. Altern. Med..

[51] Aljabali A.A.A., Bakshi H.A., Hakkim F.L., Haggag Y.A., Al-Batanyeh K.M., Zoubi M.S.A., Al-Trad B., Nasef M.M., Satija S., Mehta M. (2020). Albumin Nano-Encapsulation of Piceatannol Enhances Its Anticancer Potential in Colon Cancer Via Downregulation of Nuclear p65 and HIF-1α. Cancers.

[52] Hakkim F.L., Bakshi H.A., Khan S., Nasef M., Farzand R., Sam S., Rashan L., Al-Baloshi M.S., Abdo Hasson S.S.A., Jabri A.A. (2019). Frankincense essential oil suppresses melanoma cancer through down regulation of Bcl-2/Bax cascade signaling and ameliorates heptotoxicity via phase I and II drug metabolizing enzymes. Oncotarget.

[53] Khan M.N., Haggag Y.A., Lane M.E., McCarron P.A., Tambuwala M.M. (2018). Polymeric Nano-Encapsulation of Curcumin Enhances its Anti-Cancer Activity in Breast (MDA-MB231) and Lung (A549) Cancer Cells Through Reduction in Expression of HIF-1α and Nuclear p65 (Rel A). Curr. Drug Deliv..

[54] Bakshi H.A., Mishra V., Satija S., Mehta M., Hakkim F.L., Kesharwani P., Dua K., Chellappan D.K., Charbe N.B., Shrivastava G. (2019). Dynamics of Prolyl Hydroxylases Levels During Disease Progression in Experimental Colitis. Inflammation.

[55] Bakshi H.A., Hakkim F.L., Smitha S. (2016). Assessment of *in vitro* cytotoxicity of saffron (Crocus sativus L.) on cervical cancer cells (HEp-2) and their *in vivo* pre-clinical toxicity in normal swiss albino mice. Int. J. Herbal Med..

[56] Skemiene K., Rakauskaite G., Trumbeckaite S., Liobikas J., Brown G.C., Borutaite V. (2013). Anthocyanins block ischemia-induced apoptosis in the perfused heart and support mitochondrial respiration potentially by reducing cytosolic cytochrome c. Int. J. Biochem. Cell Biol..

[57] Bieging K.T., Mello S.S., Attardi L.D. (2014). Unravelling mechanisms of p53-mediated tumour suppression. Nat. Rev. Cancer.

[58] Grochola L.F., Taubert H., Greither T., Bhanot U., Udelnow A., Wurl P. (2011). Elevated transcript levels from the MDM2 p1 promoter and low p53 transcript levels are associated with poor prognosis in human pancreatic ductal adenocarcinoma. Pancreas.

[59] Vali F., Changizi V., Safa M. (2015). Synergistic Apoptotic Effect of Crocin and Paclitaxel or Crocin and Radiation on MCF-7 Cells, a Type of Breast Cancer Cell Line. Int. J. Breast Cancer.

[60] Mollaei H., Safaralizadeh R., Babaei E., Abedini M.R., Hoshyar R. (2017). The anti-proliferative and apoptotic effects of crocin on chemosensitive and chemoresistant cervical cancer cells. Biomed. Pharmacother..

[61] Balkhi H.M., Sana S., Haq E. (2017). Crocin induced apoptosis through p53-dependent pathway in C6 glioma Cells. Int. J. Adv. Res. Sci. Eng..

[62] Jin Y.H., Choi J., Shin S., Lee K.Y., Park J.H., Lee S.K., Yoo K.J. (2013). Panaxadiol selectively inhibits cyclin A-associated Cdk2 activity by elevating p21WAF1/CIP1 protein levels in mammalian cells. Carcinogenesis.

[63] Hanahan D., Weinberg R.A. (2000). The hallmarks of cancer. Cell.

[64] Otto T., Sicinski P. (2017). Cell cycle proteins as promising targets in cancer therapy. Nat. Rev. Cancer.

[65] Grana X., Reddy E.P. (1995). Cell cycle control in mammalian cells: Role of cyclins, cyclin dependent kinases (CDKs), growth suppressor genes and cyclin-dependent kinase inhibitors (CKIs). Oncogene.

[66] Ellinger-Ziegelbauer H., Kelly K., Siebenlist U. (1999). Cell cycle arrest and reversion of Ras-induced transformation by a conditionally activated form of mitogen-activated protein kinase kinase kinase 3. Mol. Cell Biol..

[67] Bulavin D.V., Saito S., Hollander M.C., Sakaguchi K., Anderson C.W., Appella E., Fornace A.J. (1999). Phosphorylation of human p53 by p38 kinase coordinates N-terminal phosphorylation and apoptosis in response to UV radiation. EMBO J..

[68] Keller D., Zeng X., Li X., Kapoor M., Iordanov M.S., Taya Y., Lozano G., Magun B., Lu H. (1999). The p38MAPK inhibitor SB203580 alleviates ultraviolet-induced phosphorylation at serine 389 but not serine 15 and activation of p53. Biochem. Biophys. Res. Commun..

[69] Bretones G., Delgado M.D., Leon J. (2015). Myc and cell cycle control. Biochim. Biophys. Acta.

[70] Shachaf C.M., Felsher D.W. (2005). Tumor dormancy and MYC inactivation: Pushing cancer to the brink of normalcy. Cancer Res..

[71] Zheng F.M., Long Z.J., Hou Z.J., Luo Y., Xu L.Z., Xia J.L., Liu J.W., Wang X., Kamran M., Yan M. (2014). A novel small molecule aurora kinase inhibitor attenuates breast tumor-initiating cells and overcomes drug resistance. Mol. Cancer Ther..

[72] Wang D., Dubois R.N. (2010). Eicosanoids and cancer. Nat. Rev. Cancer.

[73] Kamei D., Murakami M., Sasaki Y., Nakatani Y., Majima M., Ishikawa Y., Ishii T., Uematsu S., Akira S., Hara S. (2009). Microsomal prostaglandin E synthase-1 in both cancer cells and hosts contributes to tumour growth, invasion and metastasis. Biochem. J..

[74] Dubois R.N., Abramson S.B., Crofford L., Gupta R.A., Simon L.S., Van-De-Putte L.B., Lipsky P.E. (1998). Cyclooxygenase in biology and disease. FASEB J..

[75] Capdevilla J., Marnett L.J., Chacos N., Prough R.A., Estabrook R.W. (1982). Cytochrome P-450-dependent oxygenation of arachidonic acid to hydroxyeicosatetraenoic acids. Proc. Natl. Acad. Sci. USA.

[76] Taysi S., Koc M., Buyukokuroglu M.E., Altinkaynak K., Sahin Y.N. (2003). Melatonin reduces lipid peroxidation and nitric oxide during irradiation-induced oxidative injury in the rat liver. J. Pineal Res..

[77] Blum J., Fridovich I. (1985). Inactivation of glutathione peroxidase by superoxide radicals. Arch. Biochem. Biophys..

