# Correction: Bakshi et al. Dietary Crocin is Protective in Pancreatic Cancer while Reducing Radiation-Induced Hepatic Oxidative Damage. *Nutrients* 2020, *12*, 1901

**DOI:** 10.3390/nu17061083

**Published:** 2025-03-20

**Authors:** Hamid A. Bakshi, Mazhar S Al Zoubi, Hakkim L. Faruck, Alaa A A Aljabali, Firas A. Rabi, Amin A. Hafiz, Khalid M Al-Batanyeh, Bahaa Al-Trad, Prawej Ansari, Mohamed M. Nasef, Nitin B. Charbe, Saurabh Satija, Meenu Mehta, Vijay Mishra, Gaurav Gupta, Salem Abobaker, Poonam Negi, Ibrahim M. Azzouz, Ashref Ali K Dardouri, Harish Dureja, Parteek Prasher, Dinesh K. Chellappan, Kamal Dua, Mateus Webba Da Silva, Mohamed El Tanani, Paul A. McCarron, Murtaza M. Tambuwala

**Affiliations:** 1 School of Pharmacy and Pharmaceutical Science, Ulster University, Coleraine BT52 1SA, UK; mm.webba-da-silva@ulster.ac.uk (M.W.D.S.); p.mccarron@ulster.ac.uk (P.A.M.); 2 Department of Basic Medical Sciences, Faculty of Medicine, Yarmouk University, Irbid 566, Jordan; mszoubi@yu.edu.jo; 3 Department of Mathematics and Sciences, College of Arts and Applied Sciences, Dhofar University, Salalah 211, Oman; 4 Department of Pharmaceutics and Pharmaceutical Technology, Faculty of Pharmacy, Yarmouk University, Irbid 566, Jordan; alaaj@yu.edu.jo; 5 Department of Clinical Sciences, Faculty of Medicine, Yarmouk University, Irbid 21163, Jordan; 6 Department of Clinical Nutrition, Faculty of Applied Medical Sciences, Umm Al-Qura University, Makkah 21421, Saudi Arabia; aahafiz@uqu.edu.sa; 7 Department of Biological Sciences, Faculty of Science, Yarmouk University, Irbid 566, Jordan; albatynehk@yu.edu.jo (K.M.A.-B.); bahaa.tr@yu.edu.jo (B.A.-T.); 8 School of Biomedical Sciences, Ulster University, Coleraine BT52 1SA, UK; ansari-p@ulster.ac.uk; 9 Department of Pharmacy and Biomedical Sciences, School of Applied Sciences, University of Huddersfield, Queensgate, Huddersfield HD13DH, UK; mohamednasef103@gmail.com; 10 Departamento de Química Orgánica, Facultad de Química y de Farmacia, Pontificia Universidad Católica de Chile, Av. Libertador Bernardo O’Higgins, Santiago 340, Región Metropolitana, Chile; nitinunimi@gmail.com; 11 School of Pharmaceutical Sciences, Lovely Professional University, Phagwara, Punjab 144411, India; saurabh.21958@lpu.co.in (S.S.); vijaymishra2@gmail.com (V.M.); 12 Discipline of Pharmacy, Graduate School of Health, University of Technology Sydney, Ultimo, NSW 2007, Australia; kamal.dua@uts.edu.au; 13 School of Pharmacy, Suresh Gyan Vihar University, Jagatpura, Mahal Road, Jaipur, Rajasthan 302017, India; gauravpharma25@gmail.com; 14 Department of Gynecology, European Competence Center for Ovarian Cancer, Campus Virchow, Klinikum Charite-Universitatmedizin Berlin, Augustenburger Platz 1, 13353 Berlin, Germany; 15 School of Pharmaceutical Sciences, Shoolini University, Bajhol, Sultanpur, Solan, Himachal Pradesh 173229, India; poonam.546@shooliniuniversity.com; 16 Department of Dermatology, Venerology, and Allergology, Charite-Universitatsmedizin Berlin, Corporate Member of Freie Universitat Berlin, Chariteplatz1, 10117 Berlin, Germany; 17 Department of Forensic Science, School of Applied Sciences, University of Huddersfield, Huddersfield HD13DH, UK; drdory_02@yahoo.com; 18 Department of Pharmaceutical Sciences, Maharshi Dayanand University, Rohtak, Haryana 124001, India; harishdureja@gmail.com; 19 Department of Chemistry, University of Petroleum & Energy Studies, Dehradun 248007, India; parteekchemistry@gmail.com; 20 Department of Life Sciences, School of Pharmacy, International Medical University, Kuala Lumpur 57000, Malaysia; dinesh_kumar@imu.edu.my; 21 Pharmacological and Diagnostic Research Centre, Faculty of Pharmacy, Al-Ahliyya Amman University, Amman 19328, Jordan; m.tanani@ammanu.edu.jo

In the original publication [1], the email address of Murtaza M. Tambuwala has been updated. 

There was a mistake in Figure 3 as published. The wrong beta actin blot was used while creating Figure 3. The corrected Figure 3 appears below. The authors state that the scientific conclusions are unaffected. This correction was approved by the Academic Editor. The original publication has also been updated.

## Figures and Tables

**Figure 3:**
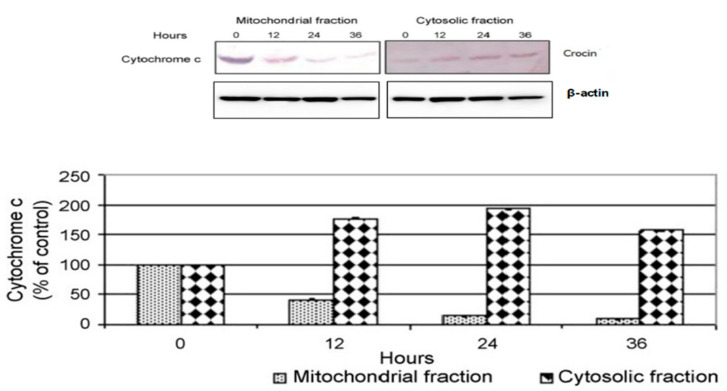
Crocin induced release of mitochondrial cytochrome c in BXPC3 cells. Release of cytochrome c from mitochondria to the cytosol was detected in BxPC-3 cells treated with crocin (10 µg/mL) at a time point of 0, 12, 24 and 36 h. The protein bands were subsequently quantified by densitometric analysis with that of control being 100% as shown just below the immunoblot data. Data represented the mean ± SEM of three independent experiments.
